# Socioeconomic inequalities in hidden hunger, undernutrition, and overweight among under-five children in 35 sub-Saharan Africa countries

**DOI:** 10.1186/s42506-019-0034-5

**Published:** 2020-03-17

**Authors:** Michael Ekholuenetale, Godson Tudeme, Adeyinka Onikan, Charity E. Ekholuenetale

**Affiliations:** 1 Department of Epidemiology and Medical Statistics, Faculty of Public Health, College of Medicine, University of Ibadan, Ibadan, Nigeria; 2 School of Medicine, College of Medical Sciences, University of Benin, Benin City, Nigeria; 3 Management Sciences for Health, Block B, Plot 564/565 Independence Avenue, Central Business District, FCT, Abuja, Nigeria; 4 Department of Economics, Benin Study Center, National Open University of Nigeria, Abuja, Nigeria

**Keywords:** Anemia, Mortality, Overweight, Stunting, Underweight, Wasting

## Abstract

**Background:**

Many underlying factors are assumed to contribute to the disparities in magnitude of childhood malnutrition. Notwithstanding, socioeconomic inequalities remain key measures to determine chronic and hidden hunger among under-five children. This study was undertaken to explore childhood malnutrition problems that are associated to household wealth-related and mother’s educational attainment in sub-Saharan Africa (SSA).

**Methods:**

Secondary data from birth histories in 35 SSA countries was used. The Demographic and Health Survey (DHS) data of 384,747 births between 2008 and 2017 in 35 countries was analyzed. The outcome variables of interest were mainly indicators of malnutrition: stunting, underweight, wasting, overweight, anemia, and under-five children survival. Household wealth-related and mother’s educational level were the measures of socioeconomic status. Concentration index and Lorenz curves were the main tools used to determine inequalities for nutritional outcomes. The statistical significance level was determined at 5%.

**Results:**

Based on the results, Burundi (54.6%) and Madagascar (48.4%) accounted for the highest prevalence of stunted children. Underweight children were 32.5% in Chad and 35.5% in Niger. Nigeria (16.6%) and Benin (16.4%) had the highest burdens of wasted children. Overall, overweight and under-five survival were significantly more in the higher household wealth, compared with the lower household wealth (Conc. Index = 0.0060; *p* < 0.001 and Conc. Index = 0.0041; *p* = 0.002 respectively). Conversely, stunting (Conc. Index = − 0.1032; *p* < 0.001), underweight (Conc. Index = − 0.1369; *p* < 0.001), wasting (Conc. Index = − 0.0711; *p* < 0.001), and anemia (Conc. Index = − 0.0402; *p* < 0.001) were significantly lower in the higher household wealth status, compared with the lower household wealth groups. Furthermore, under-five children survival was significantly more from mothers with higher educational attainment, compared with children from mothers with lower educational attainment (Conc. Index = 0.0064; *p* < 0.001). Conversely, stunting (Conc. Index = − 0.0990; *p* < 0.001), underweight (Conc. Index = − 0.1855; *p* < 0.001), wasting (Conc. Index = − 0.1657; *p* < 0.001), overweight (Conc. Index = − 0.0046; *p* < 0.001), and anemia (Conc. Index = 0.0560; *p* < 0.001) were significantly more among children from mothers with lower educational attainment. The test for differences between children from urban vs. rural was significant in stunted, underweight, overweight, and anemia for household wealth status. Also, the difference in prevalence between children from urban vs. rural was significant in stunted, underweight, and wasted for mother’s educational attainment.

**Conclusion and recommendations:**

Reduction in malnutrition could be achieved by socioeconomic improvement that is sustained and shared in equity and equality among the populace. Interventions which target improvement in food availability can also help to achieve reduction in hunger including communities where poverty is prevalent.

## Introduction

Childhood malnutrition is classified as undernutrition in stunting, wasting, and underweight. On the other hand, overweight is largely noted for inappropriate feeding patterns. Much of the world’s burden of malnutrition is notable in several resource-constrained settings. Children who suffer from malnutrition can be identified without special equipment or training due to associated morphological characteristics. Albeit, there are other forms of malnutrition that are less noticeable to the naked eye [1]. The World Health Organization (WHO) has estimated approximately two billion people suffer from “hidden hunger” globally [2]. This form of malnutrition tends to be hidden both to the person suffering the deficiency and to the outside world until it becomes so severe that clinical signs emerge. Promoting early childhood feeding best practices is crucial to improve the health of children [3]. A decline in malnutrition is important to achieve the Sustainable Development Goals (SDGs), especially those targeted to end poverty in all its forms everywhere (SDG 1), end hunger, achieve food security, improve nutrition, promote sustainable agriculture (SDG 2), ensure healthy lives, and promote well-being for all at all ages (SDG 3) [4, 5]. Due to the effects of childhood undernutrition, governments have made commitments to the global targets to reduce chronic undernutrition (stunting) by 40% by 2025 and also to reduce and maintain the prevalence of acute undernutrition (wasting) to less than 5% in children under 5 years of age [6].

Globally, hidden hunger afflicts a large number of people [7] and has numerous adverse effects including, poor health, retarded growth, low productivity, mental impairment, and unexpected death. Its effects on children’s health or survival can be acute, specifically from conception and up to 2 years of age or within the first 1000 days of life, leading to severe cognitive and physical consequences [8]. Worst still, lack of micronutrient can affect a child adversely. Besides, hidden hunger can hamper socioeconomic development within communities, especially in resource-constrained settings. Food and Agriculture Organization (FAO) reported that about 842 million people worldwide were unable to obtain their dietary energy needs between 2011 and 2013, compared with about 868 million people estimated between 2010 and 2012. By implication, one in eight persons worldwide has the likelihood to suffer from chronic hunger, which means lack of sufficient food for a healthy life. The staggering number of hungry people, about 827 million, dwell in poor-resource settings, where the prevalence of undernutrition is now estimated at 14.3% [7].

In spite of the efforts to reduce malnutrition, disparities across regions persist. The sub-Saharan Africa region has the highest prevalence of undernutrition, with sparse progress in recent time [7]. Hidden hunger as a type of undernutrition happens when the intake of micronutrients including vitamins and minerals are inadequate for optimal growth [9]. Poor diet remains the major factor that contributes to micronutrient deficiencies. The magnitude of malnutrition in the world today is worrisome. Nowadays, resource-constrained settings are changing from traditional diets made from less processed foods to greater energy-dense, processed, micronutrient-poor foods, which cause large increase in body weight and other non-communicable diseases. In the advent of these changes in nutrition, several countries now face the “triple burden” which includes lack of micronutrient, undernourishment, and overweight [10]. In more urbanized, higher income countries, hidden hunger co-exist with overweight or obesity when people feed more on macronutrients such as fats and carbohydrates [11]. Though it seems counter-intuitive, an overweight child can actually suffer from hidden hunger.

Chronic or hidden hunger causes about one third of childhood deaths that occur annually [12, 13]. Interventions to end hidden hunger and improve nutritional outcomes commonly target children. Considering this population, programs and interventions achieve success by improving health, nutritional status, and cognition later in life [14]. Though children are reported as the key population affected by hidden hunger, however, it impedes the health of all people. There are two mechanisms involved in accessing food which include physical and economic accessibility. The economic access could be decided by food prices, income, and access to social support. More so, the physical access could be decided by the quality and availability of infrastructure, such as roads, ports, communication, and food storage facilities, and other resources that enhance the functioning of markets [7].

There is evidence that efforts to enhance the health and well-being of children have paid-off by a reduction in childhood death by about half between 1990 and 2013 [15]. Similarly, efforts to improve childhood survival in SSA region led to a decline from 180 deaths per 1000 live births in 1990 to 83 deaths per 1000 live births in 2015 [13]. Addressing the risk factors for poor childhood health as identified in evidence-based studies [16–18] may have led to the past gains. The prominent factors identified to be responsible for poor growth and development include sub-optimal childhood feeding practices and infections [19, 20]. Several childhood feeding practices including inadequate breastfeeding or non-exclusive breastfeeding and complementary feeding that are insufficient in quality and quantity contribute to childhood malnourishment [19]. Also, infections, household poverty, caregiver neglect, or ignorance and food insecurity could have age-long adverse effect on children’s growth [19]. In light of the above, we have identified a number of studies on potential predictors of nutrition outcomes that have increased steeply over the last decades. Social support, availability and accessibility of food, and socioeconomic status are significant for nutritional behaviors [21]. In this paper, we endeavor to quantify inequalities in childhood malnutrition that are ascribable to individual and household socioeconomic status.

## Methods

### Data source

Here, we used secondary multi-country DHS data from birth histories in 35 SSA countries. Data of children born in the 5 years prior to the surveys were used and a large pooled sample of 384,747 births between 2008 and 2017 in various countries. The data is available in the public domain and accessed at http://dhsprogram.com/data/available-datasets.cfm. DHSs were based on a stratified multi-stage cluster sampling technique. The stratification strategy divides the population into groups. For instance, all DHS employ a region crossed by urban-rural stratification. The consideration of demographic differences in urban and rural areas supports the relatively independent designs in urban and rural areas and therefore the region crossed by urban-rural stratification. A multi-level stratification approach is used to divide the population into first-level strata and to subdivide the first-level strata into second-level strata, and so on. The two-level stratification in DHS is region and urban/rural stratification. DHS data are collected on vital sexual and reproductive health issues through interviewer’s administered questionnaires. A crucial component of the data collection is the maternity history; where women were asked about their birth histories. Thirty-five countries in SSA region were included based on availability of current data.

DHS are comparable household surveys that have been conducted in more than 85 countries worldwide since 1984. Though initially designed to expand on demographic, fertility and family planning data collected in the World Fertility Surveys and Contraceptive Prevalence Surveys, DHS continue to provide an important resource for the monitoring of vital statistics and population health indicators in resource-constrained settings. It collects a wide range of objective and self-reported data with a strong focus on indicators of fertility, reproductive health, maternal and child health, mortality, nutrition, and self-reported health behaviors among adults. Key advantages of the DHS include national coverage, high-quality interviewer training, standardized data collection procedures across countries and consistent content over time, allowing comparability across populations cross-sectionally and over time. Data from DHS facilitate epidemiological research focused on monitoring of prevalence, trends, and inequalities. A variety of robust observational data analysis methods have been used, including cross-sectional designs, repeated cross-sectional designs, spatial and multi-level analyses, intra-household designs and cross-comparative analyses [22].

### Variables selection and measurement

#### Outcome variable

##### Anemia

The hemoglobin levels were measured using the blood of the children collected through a finger or heel prick. Anemia was classified into; mild, moderate, severe or not anemic. The cut-off values of anemia were 10.0–10.9 g/dl (mild), 9.0–9.9 g/dl (moderate), < 9.0 g/dl (severe) and ≥ 11 g/dl (not anemic). Dichotomously, this was grouped as < 11 g/dl (anemic) vs. ≥ 11 g/dl (not anemic)

##### Childhood survival

The survival status of a child aged 0 to 59 months was measured to determine whether a child was alive vs. dead at the time of the survey.

##### Anthropometric measurement

This was calculated using the new Child Growth Standards released by the World Health Organization on 27 April 2006. *Z*-scores for weight-for-height (WHZ), height-for-age (HAZ), and weight-for-age (WAZ) were calculated using the WHO growth standards [23]. The following standard case-definitions were applied to each record:

- *Acute malnutrition/wasting*. This was calculated using weight-for-height. Children that were below − 2 SD from the reference median were classified wasted.
- *Underweight*. This was calculated based on weight-for-age, that is, a child is too thin for his/her age. Children whose weight-for-age is below − 2 SD from the reference population median were classified as underweight.
- *Overweight*. This was calculated by the weight-for-height. Children that are above + 2 SD from the reference median are considered overweight.
- *Stunting*. This was calculated based on height-for-age. *Z*-score (HAZ) below − 2 SD from the median of the WHO reference population was considered stunted or chronically malnourished.

#### Explanatory variables

##### Place of residence

This was classified as urban vs. rural.

##### Mother’s educational level

This was measured as no formal education, primary, secondary, or higher.

##### Wealth index

Wealth quintiles variable was computed by DHS using principal components analysis (PCA) to assign the wealth indicator weights. In their computation, they assigned scores and standardized the wealth indicator variable using household assets such as floor type, wall type, roof type, water source, sanitation facilities, radio, electricity, television, refrigerator, cooking fuel, furniture, and number of persons per room. Then, the factor loadings and *z*-scores were calculated. For each household, they multiplied the indicator values by the loadings and summed to produce the household’s wealth index value. The standardized *z*-score was finally used to classify the overall scores to wealth quintiles: poorest, poorer, middle, richer, and richest [24]. Household wealth quintiles and mothers’ educational attainment were used as measures of socioeconomic status similar to previous studies [25–27].

### Data analysis plan

Stata module survey (“svy”) command was used to adjust for stratification, clustering, and sampling weights in data analysis. Percentage and Chi-square test were used for summary statistics and bivariate analysis. Lorenz curves and concentration index were used to examine socioeconomic inequalities for nutritional outcomes. Concentration Index is positive when the Lorenz curve is below the line of equality indicating that the burden of chronic and hidden hunger is greater among high socioeconomic groups. Similarly, when concentration index value is negative, it shows that the burden of chronic and hidden hunger is higher among low socioeconomic groups. The urban vs. rural place of residence was used for stratified analyses. Lorenz curves and concentration index were used to compute contrast between various outcome variables [28]. Statistical significance was determined at *p* < 0.05. Data analysis was conducted using Stata Version 14 (StataCorp., College Station, TX, USA).

### Ethical consideration

This study was based on an analysis of population-based datasets that exist in the public domain and available online. The authors communicated with MEASURE DHS/ICF International and permission was granted to download and use the data. The DHS project obtained the required ethical approvals from the relevant research ethics committee in each country before the surveys were conducted. Measure DHS/ICF International approved the usage of the data for this study.

## Results

Table 1 shows that under-five mortality was lowest in Gambia (3.7%) and South Africa (3.8%) but highest in Chad (9.3%) and Sierra Leone (11.1%). Burundi (54.6%) and Madagascar (48.4%) accounted for the highest prevalence of stunted children. Furthermore, underweight children were 32.5% in Chad and 35.5% in Niger which had the highest burdens of underweight children in sub-Saharan Africa countries. Nigeria (16.6%) and Benin (16.4%) had the highest burdens of wasted children. Overweight children were majorly reported from Namibia (70.3%), Malawi (71.3%), Uganda (72.7%), and South Africa (73.5%). It was worrisome to find that majorities of children in sub-Saharan Africa countries had anemia (mild, moderate, and severe).

**Table 1:** Summary statistics from 35 SSA countries on nutrition indicators among under-five children; DHS, 2008-2017

Country	Study year	Sample size (*n*)	Stunted (%)	Under-5 death (%)	Underweight (%)	Wasted (%)	Overweight (%)	Anemia		
								Mild (%)	Moderate (%)	Severe (%)
Central SSA										
Madagascar	2008/09	12,448	48.4	5.6				30.4	19.6	0.9
Angola	2015/16	14,322	37.4	4.9	19.4	5.1	56.8	30.1	32.1	2.6
Congo	2011/12	9329	26.8	5.1	13.0	5.5	53.7	31.5	33.5	1.2
Gabon	2012	6067	22.9	5.3	8.3	4.1	48.3	29.5	32.5	1.8
Democratic Republic of Congo	2013/14	18,716	44.1	8.0	23.2	7.9	58.7	24.7	34.6	3.7
Total estimate	2008–2016	60,882	37.8	6.1	17.8	6.0	54.6	28.7	30.7	2.3
*P*			< 0.001	< 0.001	< 0.001	< 0.001	< 0.001	< 0.001	< 0.001	< 0.001
East SSA										
Burundi	2016/17	13,192	54.6	5.5	28.9	5.1	54.8	24.9	30.9	3.3
Comoros	2012	3149	27.7	4.0	14.5	11.7	30.8			
Ethiopia	2016	10,641	36.4	6.0	25.3	12.1	18.0	23.7	32.5	4.0
Kenya	2014	20,964	27.1	4.2	13.2	5.5	14.2			
Malawi	2015/16	17,286	35.2	4.8	12.0	3.2	71.3	27.0	34.4	1.8
Mozambique	2011	11,102	39.3	7.3	13.1	5.2	22.6	26.2	36.7	3.4
Rwanda	2014/15	7856	37.4	3.8	9.0	2.2	58.6	20.4	14.7	0.7
Tanzania	2015/16	10,233	33.6	5.1	13.7	4.9	15.6	27.2	29.7	1.5
Uganda	2016	15,522	28.4	5.2	10.8	3.9	72.7	24.4	27.8	2.3
Zambia	2013/14	13,457	39.6	5.5	14.9	6.3	20.1			
Total estimate	2011–2016	123,402	35.3	5.2	15.7	6.0	39.0	25.1	30.3	2.6
*P*			< 0.001	< 0.001	< 0.001	< 0.001	< 0.001	< 0.001	< 0.001	< 0.001
South SSA										
Lesotho	2014	3138	34.6	7.1	11.2	3.5	61.6	25.5	26.3	1.7
South Africa	2016	3548	25.9	3.8	5.7	2.9	73.5	25.8	32.6	2.7
Namibia	2013	5046	23.2	4.5	13.8	8.0	70.3	24.7	25.4	1.0
Zimbabwe	2015	6132	25.6	5.3	7.7	3.5	24.8	22.2	15.4	0.5
Total estimate	2013–2016	17,864	26.5	5.1	9.0	4.2	53.8	23.6	20.9	1.0
*P*			< 0.001	< 0.001	< 0.001	< 0.001	< 0.001	< 0.001	< 0.001	< 0.001
West SSA										
Sao Tome and Principe	2008/09	1931	27.6	4.1	13.3	12.3	32.9	31.5	27.9	1.0
Nigeria	2013	31,482	36.1	9.2	26.9	16.6	25.2			
Guinea	2012	7039	30.8	8.7	17.8	9.9	57.7	23.4	45.2	7.6
Niger	2012	12,558	41.9	7.6	35.5	18.3	63.0	26.1	46.0	2.8
Benin	2012	13,407	44.0	5.4	21.5	16.4	53.0	26.4	29.4	2.9
Cameroon	2011	11,732	31.6	8.5	13.7	5.4	59.8	27.8	32.3	2.0
Chad	2014/15	18,623	42.9	9.3	32.5	14.2	48.5			
Ghana	2014	5884	19.2	4.9	11.0	4.9	54.9	27.4	39.4	2.8
Burkina-Faso	2010	15,044	34.2	8.8	24.9	15.3	57.6	18.5	59.0	10.9
Cote d’Ivoire	2011/12	7776	29.8	8.8	14.6	7.1	60.0	24.9	47.0	3.5
Liberia	2013	7606	30.9	7.2	15.3	6.6	59.5			
Mali	2012/13	10,326	37.8	7.2	25.2	12.6	59.1	21.4	51.1	8.6
Senegal	2017	12,185	19.1	4.8	16.4	9.6	12.3	27.9	43.0	3.4
Sierra Leone	2013	11,938	37.7	11.1	16.0	9.4	68.8	26.2	47.6	6.6
Togo	2013/14	6979	28.3	6.4	16.8	7.3	55.2	26.0	42.4	2.6
Gambia	2013	8088	25.8	3.7	18.0	11.7	62.8	24.4	44.8	4.7
Total estimate	2010–2015	182,598	33.9	7.8	22.6	12.8	49.0	25.3	44.1	4.9
*P*			< 0.001	< 0.001	< 0.001	< 0.001	< 0.001	< 0.001	< 0.001	< 0.001

*P* value was obtained using Chi-square

Table 2 presents the percentages of stunting, underweight, wasting, overweight, anemia, and children under-5 years who survived to the date of the surveys across household wealth quintile and mother’s education. Based on the results, rural children were more stunted, underweight, wasted, and anemic across household wealth quintile and levels of mother’s education, compared with the urban children. However, urban children were more overweight and had higher under-five survival across household wealth quintile and mother levels of mother’s education, compared with their rural counterpart. Furthermore, the concentration index, quantified the degree of wealth-related and mother’s education inequalities in stunting, underweight, wasting, overweight, anemia, and under-five survival. Overall, overweight and under-five survival were significantly more in higher household wealth, compared to the lower household wealth groups, (Conc. Index = 0.0060; *p* < 0.001) and under-five survival (Conc. Index = 0.0041; *p* = 0.002), respectively. Conversely, stunting (Conc. Index = − 0.1032; *p* < 0.001), underweight (Conc. Index = − 0.1369; *p* < 0.001), wasting (Conc. Index = − 0.0711; *p* < 0.001), and anemia (Conc. Index = − 0.0402; *p* < 0.001) were significantly lower in the higher household wealth status, compared to the lower household wealth groups. Furthermore, under-five children survival was significantly more from mothers with higher educational attainment, compared with children from mothers with lower educational attainment (Conc. Index = 0.0064; *p* < 0.001). More so, stunting (Conc. Index = − 0.0990; *p* < 0.001), underweight (Conc. Index = − 0.1855; *p* < 0.001), wasting (Conc. Index = − 0.1657; *p* < 0.001), overweight (Conc. Index = − 0.0046; *p* < 0.001), and anemia (Conc. Index = 0.0560; *p* < 0.001) were significantly more among children from mothers with lower educational attainment. The test for differences between children from urban vs. rural was significant in stunting, underweight, overweight, and anemia for household wealth status.

**Table 2:** Prevalence and concentration index of stunting, underweight, wasting, overweight, anemia, under-5 mortality by household wealth quintile, and mother’s education; DHS, 2008–2017

Variable	Stunting			Underweight			Wasting			Overweight			Anemia			Under-5 survival		
	Urban	Rural	Total	Urban	Rural	Total	Urban	Rural	Total	Urban	Rural	Total	Urban	Rural	Total	Urban	Rural	Total
Household wealth quintile																		
Poorest (%)	9.4	36.9	30.7	10.5	39.8	33.4	8.4	38.5	30.4	6.0	33.1	25.2	7.0	36.2	28.4	6.3	33.6	25.4
Poorer (%)	11.0	28.6	24.6	11.1	28.4	24.6	8.5	27.6	22.4	8.4	27.6	22.0	9.1	27.8	22.8	8.4	27.6	21.9
Middle (%)	19.0	20.3	20.0	19.0	18.8	18.9	17.7	19.5	19.1	15.5	21.5	19.7	17.4	20.5	19.7	15.8	21.3	19.7
Richer (%)	28.7	11.8	15.6	28.3	11.0	14.7	28.5	12.0	16.4	26.6	13.9	17.6	28.6	12.3	16.7	27.1	13.7	17.7
Richest (%)	31.9	2.4	9.1	31.1	2.1	8.4	36.9	2.4	11.6	43.5	3.9	15.5	37.9	3.1	12.4	42.4	3.8	15.4
Overall (%)	26.1	38.3	34.6	13.5	21.2	18.9	8.1	9.6	9.1	47.8	48.9	48.5	60.5	66.6	64.9	94.3	93.1	93.5
Concentration Index value	− 0.1194	− 0.0472	− 0.1032	− 0.1304	− 0.0816	− 0.1369	− 0.0493	− 0.0581	− 0.0711	0.0144	0.0093	0.0060	− 0.0486	− 0.0264	− 0.0402	0.0031	0.0024	0.0041
SE	0.0036	0.0018	0.0017	0.0055	0.0028	0.0025	0.0074	0.0045	0.0039	0.0017	0.0011	0.0009	0.0024	0.0013	0.0012	0.0004	0.0003	0.0002
*P* value^α^	< 0.001	< 0.001	< 0.001	< 0.001	< 0.001	< 0.001	< 0.001	< 0.001	< 0.001	< 0.001	< 0.001	< 0.001	< 0.001	< 0.001	< 0.001	< 0.001	< 0.001	< 0.001
Urban-rural comparison																		
*z*-stat	17.79			7.90			− 1.02			− 2.53			8.19			− 1.57		
Difference in concentration index values	0.0721			0.0489			− 0.0088			− 0.0051			0.0222			− 0.0008		
*P* value^β^	< 0.001			< 0.001			0.309			0.012			< 0.001			0.116		
Mother’s educational level																		
None (%)	29.9	52.2	47.1	37.3	62.2	56.8	36.4	64.9	57.3	26.3	48.3	41.9	29.5	53.9	47.4	24.9	47.8	40.9
Primary (%)	35.5	36.9	36.6	31.8	29.0	29.6	27.5	24.7	25.4	28.7	37.0	34.6	31.4	33.7	33.1	30.1	37.1	35.0
Secondary (%)	31.3	10.5	15.3	28.0	8.5	12.7	31.3	9.9	15.6	38.8	14.1	21.3	35.3	12.1	18.3	38.2	14.3	21.5
Higher (%)	3.3	0.3	1.0	2.9	0.3	0.8	4.8	0.5	1.7	6.1	0.7	2.2	3.8	0.4	1.3	6.8	0.9	2.6
Overall (%)	26.1	38.3	34.6	13.5	21.2	18.9	8.1	9.6	9.1	47.8	48.9	48.6	60.5	66.6	64.9	94.3	93.1	93.5
Concentration Index value	− 0.1184	− 0.0624	− 0.0990	− 0.1840	− 0.1539	− 0.1855	− 0.1348	− 0.1680	− 0.1657	− 0.0049	− 0.0011	− 0.0046	− 0.0549	− 0.0507	− 0.056	0.0056	0.0057	0.0064
SE	0.0036	0.0017	0.0016	0.0055	0.0026	0.0024	0.0074	0.0042	0.0037	0.0017	0.0010	0.0009	0.0023	0.0013	0.0011	0.0003	0.0002	0.0002
*P* value^α^	< 0.001	< 0.001	< 0.001	< 0.001	< 0.001	< 0.001	< 0.001	< 0.001	< 0.001	0.004	0.286	< 0.001	< 0.001	< 0.001	< 0.001	< 0.001	< 0.001	< 0.001
Urban-rural comparison																		
*z*-stat	13.97			4.94			− 3.91			1.92			1.60			0.20		
Difference in concentration index values	0.0560			0.0301			0.0332			0.0038			0.0043			0.0001		
*P* value^β^	< 0.001			< 0.001			< 0.001			0.055			0.110			0.842		

*SE* standard error; *P* value^α^ and *P* value^β^ were obtained using Concentration Index for overall inequalities across socioeconomic groups and measuring rural vs. urban differences, respectively

**Fig. 1:**
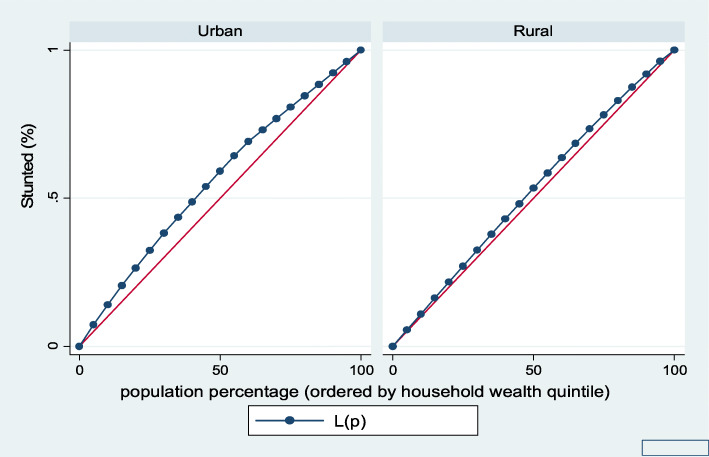
Urban-rural Lorenz curve for stunting

**Fig. 2:**
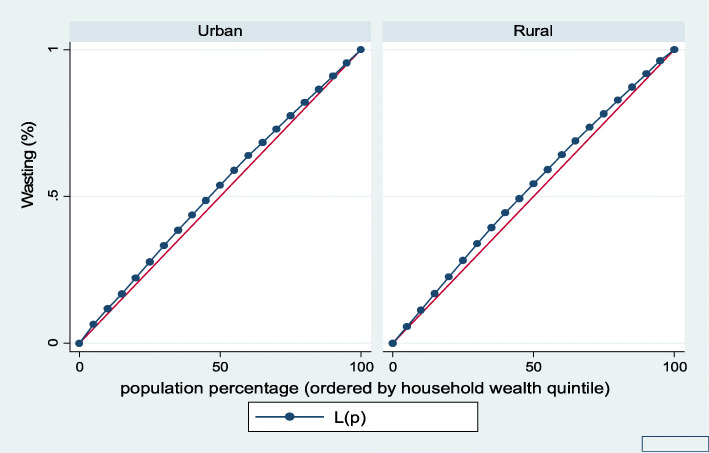
Urban-rural Lorenz curve for wasting

**Fig. 3:**
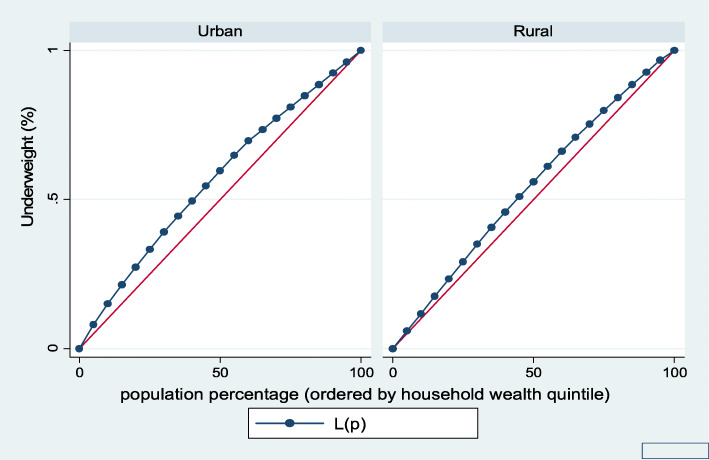
Urban-rural Lorenz curve for underweight

**Fig. 4:**
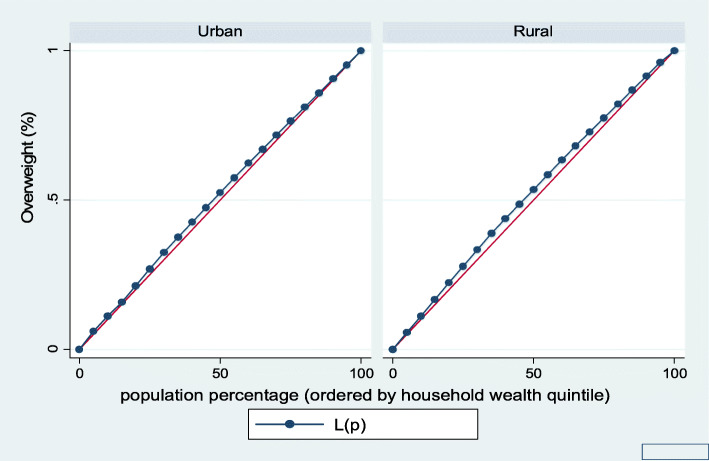
Urban-rural Lorenz curve for overweight

**Fig. 5:**
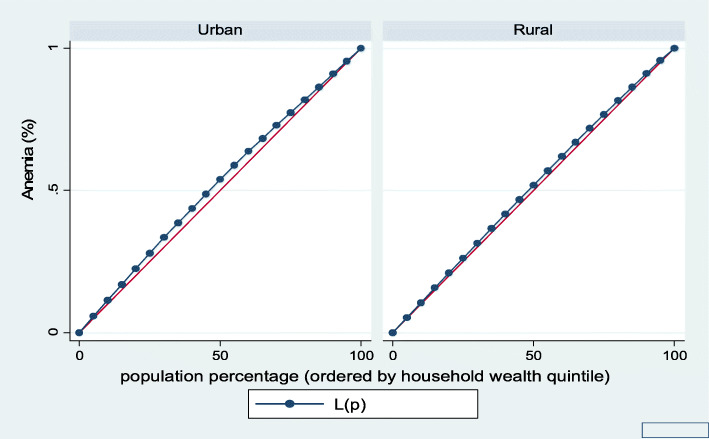
Urban-rural Lorenz curve for anemia

**Fig. 6:**
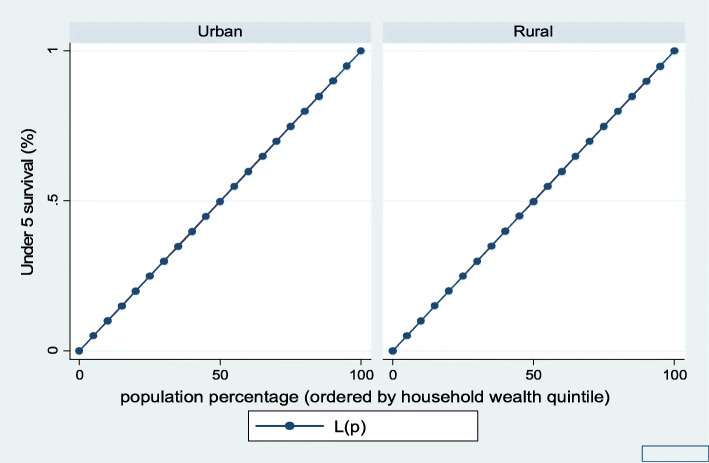
Urban-rural Lorenz curve for childhood survival

Figures 1, 2, 3, 4, 5, and 6 show the household wealth-related inequalities for the stunting, wasting, underweight, overweight, anemia, and under-five children survival. The more the Lorenz curves sags away from the line of equality, the greater the degree of inequality. The inequalities in household wealth level were more among stunted, underweight, and anemic urban children, as the areas between the curve and the line of inequality was maximal. This is consistent with the results obtained from the concentration index model (− 0.0036, − 0.1304, and − 0.0486, respectively).

**Fig. 7:**
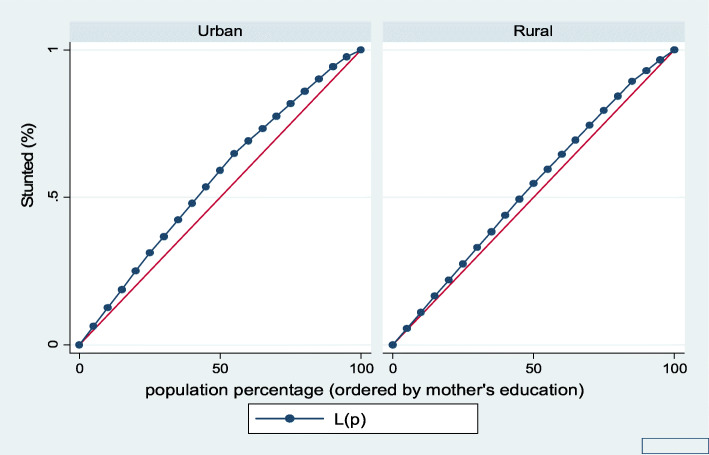
Urban-rural Lorenz curve for stunting

**Fig. 8:**
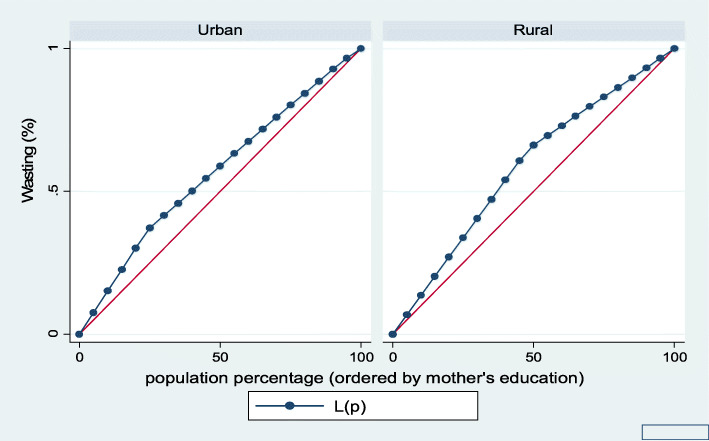
Urban-rural Lorenz curve for wasting

**Fig. 9:**
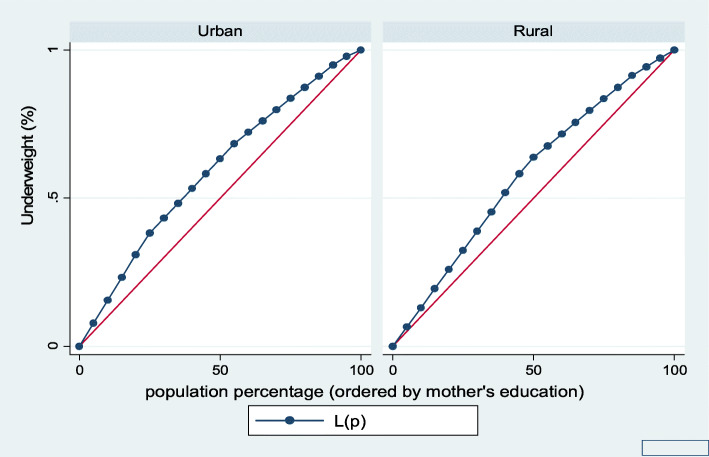
Urban-rural Lorenz curve for underweight

**Fig. 10:**
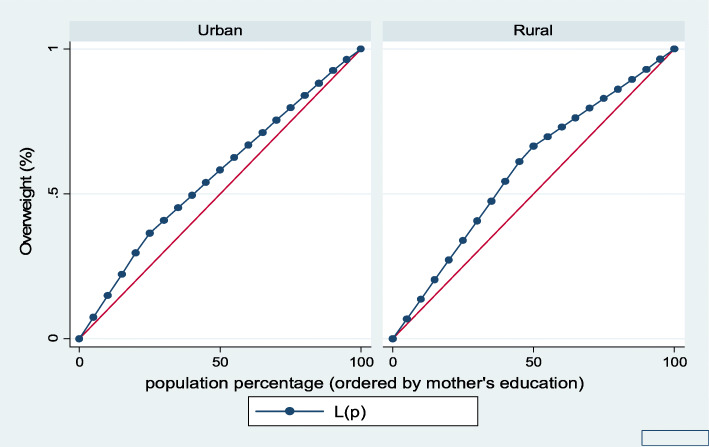
Urban-rural Lorenz curve for overweight

**Fig. 11:**
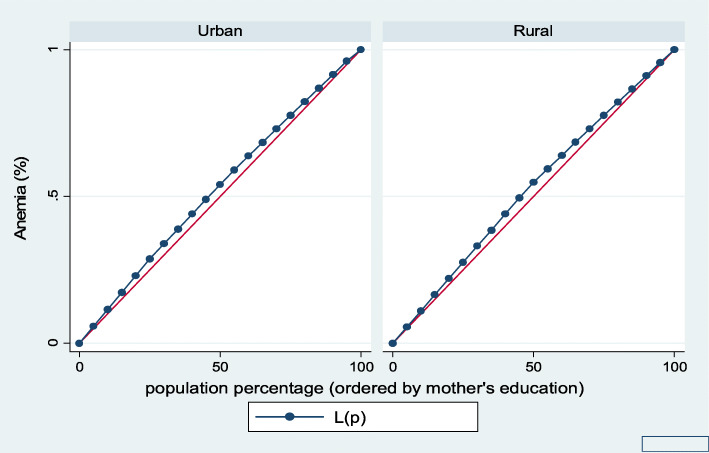
Urban-rural Lorenz curve for anemia

**Fig. 12:**
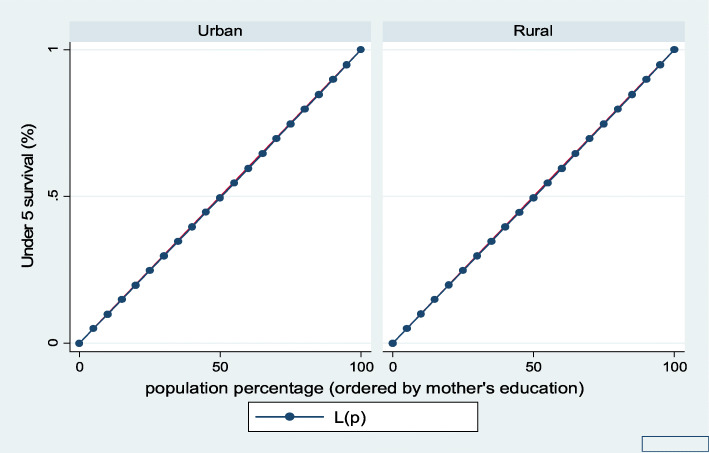
Urban-rural Lorenz curve for childhood survival

Figures 7, 8, 9, 10, 11, and 12 show mothers’ educational attainment inequalities for the stunting, wasting, underweight, overweight, anemia, and under-five children survival. The farther the Lorenz curves draw away from the line of equality, the higher the degree of inequality. The inequalities in mother’s educational attainment varied among children by nutritional indicators, as the areas between the curve and the line of inequality was maximal. For example, comparing with the outcome of the concentration index, overall, stunting (− 0.0990), underweight (− 0.1855), wasting (− 0.1657), overweight (− 00046), and anemia (− 0.0560) were higher among children from lower mother’s education.

## Discussion

The findings in the paper suggest that sub-Saharan Africa countries have disparities on several indicators of malnutrition including stunting, underweight, wasting, overweight, anemia, and childhood survival. Interestingly, this paper contributes to this growing empirical literature on household wealth-related and maternal education inequalities in child malnutrition. In this study, we investigated that socioeconomic inequalities in malnutrition is present in sub-Saharan African children. The key findings indicated that the disadvantaged have more burdens from malnutrition, and that the resultant inequalities were higher for other malnutrition indicators except for childhood survival. There is consistency of this results with previous evidence that reported that socioeconomic status has effect on the conditions that precipitated malnourishment [29–31]. By using concentration index and Lorenz curves to examine socioeconomic inequalities in childhood malnutrition, to draw viable conclusions about regional inequalities in malnutrition makes this paper very unique.

Furthermore, inequalities in mother’s education and indicators of malnutrition were a notable finding in this study. This is in line with reports from a previous study [32]. The contribution of mother’s education to the inequalities in childhood malnutrition could be due to differences in the knowledge or ability of mothers to decide proper nutrition for children in terms of balanced diets and accessibility to food commodities. This factor is a key determinant associated with inequality in childhood malnutrition. Previous studies have reported the function of mother’s education in malnutrition, either by investigating inequalities [33, 34] or by measuring malnutrition and its associated factors [35, 36].

### Strengths and limitations

The major strength was the use of multi-country large data sets which provide generalizable estimates. In addition, the data allowed large reporting of the prevalence of chronic and hidden hunger among under-five children. However, using a cross-sectional study, we collected data across a number of countries at different points in time. Therefore, the distribution of nutritional outcomes could have changed over time. Also, DHS does not collect data on household income or expenditure, which are the traditional indicators used to measure wealth. The assets-based wealth index used here is only a proxy indicator for household economic status, and it does not always produce results similar to those obtained from direct measurements of income and expenditure where such data are available or can be collected reliably.

## Conclusion and recommendations

To end the problems of childhood malnutrition, improvements in maternal education and household wealth status are required, especially initiatives that go beyond the limits of the medical domain. Proper nutrition, resulting in healthy growth would help to reduce chronic and hidden hunger and their associated morbidity and mortality, particularly among the poor and the rural dwellers. In resource-constrained settings, reduction in hunger could be achieved with socioeconomic improvement that is not only sustained, but also shared in equity and equality among the populace. Interventions which target improvement in food availability and agricultural productivity can also help to achieve hunger reduction. When these combine with social protection and various measures that increase the incomes of the disadvantaged families to acquire food commodities, they can have more positive effect and instigate rural development, through creation of active markets and employment opportunities, leading to equitable economic growth at large. Ministries, agencies, and non-governmental organizations should form an alliance to improve food security and develop policies or programs to address the issue of hunger. Women’s empowerment should be encouraged as a tool to address chronic and hidden hunger in sub-Saharan Africa.

## Data Availability

Data sources were from Demographic and Health surveys (DHS) and available here: http://dhsprogram.com/data/available-datasets.cfm
